# Association of Exposure to Violence at Home with Aggressive Behaviors and Depressive Symptoms in Adolescence: Findings from the Generation XXI Cohort

**DOI:** 10.1007/s40653-026-00929-9

**Published:** 2026-06-11

**Authors:** Mariana Amorim, Assumpta Nwodu, Sílvia Fraga

**Affiliations:** EPIUnit ITR, Instituto de Saúde Pública da Universidade do Porto, Universidade do Porto, Porto, Portugal

**Keywords:** Domestic violence, Bullying, Cyberbullying, Depressive symptoms, Adolescence

## Abstract

Objective: This study aimed to examine the association between exposure to violence at home and aggressive behaviors and depressive symptoms in adolescence. Methods: This cross-sectional study used data from the Portuguese cohort Generation XXI (n = 4,640). At age 13, participants completed self-report questionnaires assessing exposure to violence at home, involvement in physical fights, experience of bullying and cyberbullying, and depressive symptoms. Logistic regression models were used to estimate associations between witnessing violence at home and aggressive behaviors and depressive symptoms, adjusting for sex, maternal education, parental unemployment, family structure, and direct victimization, and child psychological and physical aggression. Results: Overall, 14% of participants reported witnessing violence at home, 10.1% experienced psychological aggression, and 9.7% reported physical abuse. Involvement in fights was reported by 17.4%, experience of bullying by 21.3%, and cyberbullying by 4.6%; 12.4% reported depressive symptoms. Witnessing violence at home was significantly associated with all outcomes. A dose–response pattern was found for involvement in fights (AOR_1−2 acts_: 2.01, 95%CI: 1.53–2.64; AOR_≥ 3 acts_: 3.33, 95%CI: 1.88–5.92) and depressive symptoms (AOR_1−2 acts_: 1.97, 95%CI: 1.43–2.72; AOR_≥ 3 acts_: 2.22, 95%CI: 1.11–4.44). Exposure to three or more acts of violence was also associated with experience of bullying (AOR: 2.07, 95%CI: 1.17–3.67) and cyberbullying (AOR: 3.74, 95%CI: 1.76–7.96). Conclusions: Witnessing violence at home has a detrimental impact on adolescent behavior and emotions. Findings highlight the urgent need for targeted interventions and policies to prevent domestic violence and mitigate its effects on children’s health and development.

## Introduction

Domestic violence is defined by all acts of physical, sexual, psychological or economic violence that occurs within the family or domestic unit or between former or current spouses or partners (Council of Europe, 2011). Beyond its immediate harm, domestic violence often reinforces cycles of aggression and trauma, contributing to the intergenerational transmission of violent behaviors and the perpetuation of dysfunctional family dynamics (Herrenkohl et al., 2013).

Globally, one in six children is exposed to physical domestic and family violence before the age of 18, though prevalence rates vary across regions (Whitten et al., 2024). In Portugal, the issue remains pressing, considering that in 2024, 76% of the 31,242 crimes reported to the Portuguese Victim Support Association (APAV) were related to domestic violence (Associação Portuguesa de Apoio à Vítima (APAV), 2025). It is important to note that these figures only capture cases in which victims chose to disclose the violence and seek support, suggesting that the magnitude of the problem may be underestimated.

In Portugal, domestic violence crimes encompass intimate partner violence, as well as violence against children and older people, along with other forms of violence that may occur in the domestic setting. However, witnessing intimate partner violence is not considered a crime against children, as they are not regarded as being directly attacked. Some authors argue that distinctions should not be made between children who directly experience violence and those who witness violence between their parents at home, as they are victims in both cases (Bacchini & Esposito, 2020; Rydström et al., 2019).

There is evidence that children growing up in homes with violence have more psychological, social, and academic problems than children growing up in non-violent homes, even when the children have not been subjected to violence themselves (Sternberg et al., 2006). Although differences in the effects of self-experiencing versus witnessing violence at home may exist, the consequences of witnessing violence should not be underestimated. According to the World Health Organization, witnessing physical violence in the home during childhood, defined as hearing or seeing a parent punch, kick or beat the other parent, their boyfriend or girlfriend, or brothers or sisters, is a global concern (World Health Organization, 2020). Studies have consistently shown that children who witness interparental violence may experience similar levels of internalizing and externalizing problems as those who are directly abused (Kitzmann et al., 2003; Sternberg et al., 2006), supporting that witnessing violence at home can be as harmful as being a direct victim of violence.

Growing up in a home with violence may be a terrifying and traumatic experience that can impact every aspect of a child’s life, growth, and development and cause long-term health effects. Consequently, they may exhibit externalizing behaviors such as aggression, conduct problems, and antisocial traits (Margolin & Gordis, 2000; Moylan et al., 2010; Smith et al., 2008), which may later interfere with their ability to establish and maintain healthy interpersonal relationships (Chen & Lee, 2021; Evans et al., 2008; Lakhdir et al., 2020). Exposure to family violence may also influence how children interact with peers, increasing the likelihood of involvement in violent or conflictual relationships outside the family context. Children exposed to violent family environments may internalize aggressive norms and reproduce them in peer interactions, which can manifest as involvement in aggressive behaviours during adolescence (Baldry, 2003; Holt et al., 2008). Evidence suggests that adverse family experiences, including exposure to domestic violence and negative parenting behaviours, are associated with a higher risk of both bullying perpetration and victimization, highlighting the role of family environments in shaping aggressive peer dynamics (Holt et al., 2008; Lereya et al., 2015). More recently, attention has turned to the extension of these dynamics to digital environments, where cyberbullying has emerged as a significant form of peer aggression among adolescents and has been linked to broader patterns of interpersonal violence and normative beliefs about aggression (Kowalski et al., 2019).

This study aims to examine the prevalence of exposure to violence at home in a sample of children and to investigate its association with aggressive behaviors (involvement in fights, bullying and cyberbullying) and depressive symptoms during adolescence. In doing so, this study intends to contribute to the expanding body of research demonstrating that witnessing violence is far from harmless. It seeks to provide a foundation for re-evaluating the concept of exposure to violence and, consequently, to advocate for a reassessment of the legal framework in order to ensure greater attention to and protection of children’s well-being.

## Methods

### Study Design and Participants

This is a cross-sectional study based on data from the Portuguese population-based cohort Generation XXI. A total of 8,495 mothers, who gave birth to 8,647 live-born infants, were enrolled into the cohort. Recruitment occurred between April 2005 and September 2006 across all five public maternity units covering six municipalities of the metropolitan area of Porto, Portugal. All the maternities, except one, are included in a general hospital, and all correspond to level III maternity units, with differentiated perinatal support. At birth, 91.4% of the invited mothers accepted to participate (Alves et al., 2012; Larsen et al., 2013). Participants were first evaluated shortly after birth, and followed-up at the ages 4, 7, 10 and 13 years (86%, 80%, 74% and 54% participation rate, respectively). In all study waves, information on socio-economic and demographic characteristics, obstetric history, history of disease, and health-related behaviors was collected by trained interviewers using structured questionnaires. The study protocol was approved by Ethics Committees of the Faculty of Medicine of the University of Porto/S. João Hospital and the Institute of Public Health of the University of Porto. A signed informed consent according to the principles of the Declaration of Helsinki was required from all participants and signed by the legal guardian. This study analysed data from participants at age 13 years (*n* = 4,640).

### Data Collection

#### Witnessing Violence at Home

Witnessing violence at home was assessed by asking participants if the adults that live in their homes (father, mother, grandparents, stepparents) at some point: (1) threatened to hurt someone and it seemed like they could do it; (2) during an argument, did they break or damage something, punch or throw something against the wall; (3) someone pushes another person; (4) someone hit or slap another person; (5) someone gets kicked, choked, or beaten up. The number of acts of violence was calculated by summing the acts participants reported (ranging from 0 to 5) and three categories were considered 0, 1–2 and 3 or more.

#### Aggressive Behaviors

Involvement in fights was assessed by asking participants how many times they were involved in a physical fight in the last 12 months, using an 8-point Likert scale from never to 12 or more times. Participants were considered being involved in fights if they answered at least once and not being involved if they answered none.

Information on bullying was assessed by asking participants two questions: “Have you ever suffered from or been a victim of bullying?” and “Have you ever done that to someone?”. Similarly, information on cyberbullying was assessed by asking participants if they ever suffered from or been a victim of cyberbullying and if they ever teased or made fun of someone through the internet or phone or posted something online without that person’s consent. Participants experiencing bullying and cyberbullying were categorized as “victim,” when they answered “yes” to the question regarding victimization, but answered “no” to the aggressive question; participants were classified as a “bully” when answered “yes” to the aggressive question, but answered “no” to the victimization question; or as “bully-victim” when was involved both as a victim and as bully simultaneously. For analytical purposes, a dichotomous variable was created to reflect any experience of bullying and cyberbullying – a participant was considered experiencing these behaviors if they answered “yes” to at least one of the two questions and not experiencing if they answered “no” to the two questions.

#### Depressive Symptoms

Depressive symptoms were measured using the validated Portuguese version of the self-reported Beck Depression Inventory-II. A score cut-off of 13 was used to define the presence of relevant depressive symptoms (Campos & Gonçalves, 2011; Coelho et al., 2002).

#### Other Covariates

Participants reported the experience of adverse childhood experiences (ACEs) by answering a self-administered questionnaire that included 15 questions about childhood physical and psychological abuse and exposure to forms of household dysfunction (Felitti et al., 1998; Finkelhor et al., 2013). For the purpose of this study, we used two items of the questionnaire to assess if the adolescent was a victim of psychological (Did someone in the household swear at, insult, put you down, or humiliate you? ) and/or physical aggression (Did someone in the household hit, kick, or punch you? ).

Parents reported employment status, family structure, education, and household income. Parental education was measured as the number of years of formal schooling and categorized as 9 years or less, 10–12 years, and > 12 years. The household income included salaries and other sources and was categorized into three categories. The lowest category was defined as having equal to or less than €1,000 disposable per month, which represented a situation of both parents receiving less than the national minimum wage of €580, before taxes, in 2018 (PORDATA). The intermediate category was defined as between €1,001 and €2,000 and the highest category > €2,000 per month. Unemployment status was used to assess whether one or both parents were unemployed. Data on family structure was assessed by asking participants whom the child lives with: mother and father, only mother or father, or neither mother nor father.

### Statistical Analysis

The chi-square test or Fisher exact test were used to compare proportions, as appropriate. Logistic regression analyses were performed to identify the association between witnessing domestic violence and aggressive behaviors (fights, bullying and cyberbullying), as well as depressive symptoms. We calculated crude (OR, 95% CI) and adjusted odds ratios, with 95% confidence intervals, (AOR, 95% CI) for co-variates such as sex, maternal education, parent’s unemployment, family structure and child’s experience of psychological and physical violence. All statistical analysis was performed using Statistical Package for the Social Science version 27.0 (SPSS).

### Results

Sociodemographic characteristics of participants at age 13 years are presented in Table 1. Participants are mainly boys, with mothers and fathers with 9 or less years of education, employed, living with mother and father and in households with €1,001– €2,000 of monthly income.

**Table 1:** Sociodemographic characteristics of participants at age 13 years (*n* = 4,640)

Sociodemographic characteristics	*n* (%)
Child’s sex	
Girl	2246 (48.4)
Boy	2394 (51.6)
Maternal education (years)	
≤ 9	1539 (34.7)
10–12	1437 (32.4)
> 12	1459 (32.9)
Paternal education (years)	
≤ 9	1211 (35.0)
10–12	1114 (32.2)
> 12	1137 (32.8)
Parental unemployment	
None of the parents	2763 (78.6)
One of the parents	718 (20.4)
Both parents	33 (0.9)
Household monthly income (€)	
≤ 1000	773 (17.4)
1001–2000	2175 (49.0)
> 2000	1494 (33.6)
Family structure	
Living with mother and father	3421 (74.3)
Living with only mother or father/neither mother nor father	1181 (25.7)

Note: The total may not add 4,640 due to missing values

Results show that 11.8% of participants reported witnessing 1–2 acts of violence, while 2.2% reported witnessing 3 or more acts of violence at home. Specifically, 6.6% saw someone push another person, 5.6% witnessed someone hitting or slapping another person, and 5.4% observed an argument where an object was broken, punched, or thrown against the wall. Boys reported witnessing all types of domestic violence more frequently than girls, except for witnessing someone break or damage something during an argument (Table 2). Regarding the experience of violence, 10.1% of participants reported being victims of psychological aggression and 9.7% reported experiencing physical violence, with more boys than girls reporting such experiences (10.6% vs. 8.8%). For aggressive behaviors, 17.4% of participants reported being involved in physical fights, 21.3% in bullying, and 4.6% in cyberbullying. Boys and girls revealed different patterns of involvement. More boys than girls reported being involved in physical fights, (25.9% vs. 8.5%). However, girls reported more frequently experiences of bullying as victims (21.9% vs. 16.7%), while boys reported being cyberbullies more often than girls (2.9% vs. 0.7%). In addition, 12.4% of participants reported significant depressive symptoms, with higher proportions in girls than in boys (18.5% vs. 6.7%) (Table 2).

**Table 2:** Prevalence of witnessing and experiencing violence at home, involvement in aggressive behaviours and depressive symptoms at age 13, by child’s sex (*n* = 4,640)

	Total *n* (%)	Girls	Boys	*P* value
Acts of witnessed violence at home				
Have they threatened to hurt someone and it seemed like they could actually do it?				
No	4349 (95.9)	2128 (96.6)	2221 (95.2)	**0.015**
Yes	188 (4.1)	75 (3.4)	113 (4.8)	
During an argument, did they break or damage something, punch or throw something against the wall?				
No	4289 (94.6)	2078 (94.5)	2211 (94.6)	0.822
Yes	246 (5.4)	121 (5.5)	125 (5.4)	
Did someone push another person?				
No	4239 (93.4)	2085 (94.6)	2154 (92.2)	**< 0.001**
Yes	299 (6.6)	118 (5.4)	181 (7.8)	
Did you see someone hit or slap another person?				
No	4274 (94.4)	2111 (96.1)	2163 (92.7)	**< 0.001**
Yes	255 (5.6)	85 (3.9)	170 (7.3)	
Did someone get kicked, choked, or beaten up?				
No	4466 (98.5)	2187 (99.3)	2279 (97.8)	**< 0.001**
Yes	66 (1.5)	15 (0.7)	51 (2.2)	
Number of witnessed acts of violence				
0	3879 (86.0)	1926 (88.0)	1953 (84.1)	
1–2	531 (11.8)	229 (10.5)	302 (13.0)	
≥ 3	99 (2.2)	33 (1.5)	66 (2.8)	**< 0.001**
Experienced violence				
Someone in my home has insulted or humiliated me				
No	4074 (89.9)	1959 (89.2)	2115 (90.6)	0.115
Yes	456 (10.1)	237 (10.8)	219 (9.4)	
Someone in my home has hit, kicked, or punched me				
No	4087 (90.3)	2003 (91.2)	2084 (89.4)	**0.036**
Yes	441 (9.7)	193 (8.8)	248 (10.6)	
Physical fights				
No	3768 (82.6)	2023 (91.5)	1745 (74.1)	**< 0.001**
Yes	796 (17.4)	187 (8.5)	609 (25.9)	
Bullying				
No experience	3582 (78.7)	1682 (76.4)	1900 (80.9)	
Victim	873 (19.2)	481 (21.9)	392 (16.7)	
Bully	46 (1.0)	19 (0.9)	27 (1.1)	
Bully-Victim	48 (1.1)	19 (0.9)	29 (1.2)	**< 0.001**
Cyberbullying				
No experience	4349 (95.4)	2119 (96.0)	2230 (94.9)	
Victim	114 (2.5)	65 (2.9)	49 (2.1)	
Bully	83 (1.8)	16 (0.7)	67 (2.9)	
Bully-Victim	11 (0.2)	7 (0.3)	4 (0.2)	**< 0.001**
Depressive symptoms				
No	3979 (87.6)	1794 (81.5)	2185 (93.3)	
Yes	564 (12.4)	408 (18.5)	156 (6.7)	**< 0.001**

Note: The total may not add 4,640 due to missing values

Participants who reported witnessing violence at home were mainly those whose mothers had lower educational levels, with both parents unemployed, lived in lower-income households, and with only one parent or none (Table 3). The same tendency was observed among adolescents who reported being involved in fights. Participants with depressive symptoms were more often those with lower educated parents, lower-income households, and who lived with only one or no parents. Furthermore, experiencing bullying was more frequently reported by those with lower educated parents, whose parents were both unemployed, who lived in households with less income, and who lived with only one or no parents. Participants who experienced psychological and physical aggression reported more frequently witnessing violence at home, experiencing aggressive behaviors and having depressive symptoms, than their counterparts (Table 3).

**Table 3:** Witnessing violence at home, aggressive behaviours and depressive symptoms at age 13, according to sociodemographic characteristics (*n* = 4,640)

Sociodemographic characteristics	Witnessing violence at home (Yes)	Physical fights (Yes)	Bullying* (Yes)	Cyberbullying* (Yes)	Depressive symptoms (Yes)
Maternal education (years)					
≤ 9	**227 (15.2)**	**317 (21.0)**	**354 (23.5)**	76 (5.0)	**218 (14.5)**
10–12	**202 (14.4)**	**236 (16.7)**	**306 (21.7)**	62 (4.4)	**169 (12.0)**
> 12	**163 (11.5)**	**196 (13.6)**	**252 (17.6)**	57 (4.0)	**139 (9.7)**
Paternal education (years)					
≤ 9	156 (13.2)	**229 (19.2)**	**247 (20.8)**	52 (4.4)	**159 (13.4)**
10–12	138 (12.7)	**167 (15.2)**	**212 (19.3)**	42 (3.8)	**117 (10.7)**
> 12	114 (10.3)	**151 (13.5)**	**184 (16.5)**	40 (3.6)	**103 (9.2)**
Parental unemployment					
None of the parents	**299 (11.0)**	**427 (15.6)**	**477 (17.5)**	**106 (3.9)**	292 (10.7)
One of the parents	**113 (16.5)**	**135 (19.4)**	**182 (26.3)**	**31 (4.5)**	92 (13.4)
Both parents	**6 (18.8)**	**8 (25.0)**	**13 (40.6)**	**5 (15.6)**	3 (9.4)
Household monthly income (€)					
≤1000	**137 (18.6)**	**158 (21.1)**	**211 (28.3)**	44 (5.9)	**118 (15.8)**
1001-2000	**298 (14.0)**	**368 (17.2)**	**439 (20.6)**	89 (4.2)	**263 (12.3)**
>2000	**163 (11.2)**	**220 (14.9)**	**256 (17.4)**	59 (4.0)	**152 (10.3)**
Family structure					
Living with mother and father	**387 (11.6)**	**535 (15.9)**	**633 (18.8)**	**132 (3.9)**	**365 (10.9)**
Living with only mother or father/neither mother nor father	**245 (21.4)**	**251 (21.7)**	**321 (27.9)**	**70 (6.1)**	**193 (16.8)**
Experiencing psychological violence					
No	**452 (11.2)**	**675 (16.6)**	**795 (19.6)**	**161 (4.0)**	**405 (10.0)**
Yes	**187 (41.2)**	**117 (25.7)**	**163 (35.8)**	**46 (10.1)**	**156 (34.3)**
Experiencing physical violence					
No	**462 (11.4)**	**662 (16.2)**	**816 (20.0)**	**161 (3.9)**	**456 (11.2)**
Yes	**177 (40.3)**	**131 (29.7)**	**143 (32.6)**	**45 (10.2)**	**103 (23.5)**

Note: The total may not add 4,640 due to missing values

Bolds represent statistically significant differences (*p* < 0.05)

*Includes any type of experience of bullying or cyberbullying

A dose–response association was found between the number of acts of violence witnessed at home and behavioural and emotional problems. Adolescents who reported witnessing one to two acts of violence had higher odds of being involved in fights (AOR: 2.01, 95%CI: 1.53–2.64) and presenting depressive symptoms (AOR: 1.97, 95%CI: 1.43–2.72), while those who witnessed three or more acts showed even higher odds for both outcomes (AOR for fights: 3.33, 95%CI: 1.88–5.92; AOR for depressive symptoms: 2.22, 95%CI: 1.11–4.44). These associations remained significant after adjusting for sex, maternal education, parental unemployment, family structure, and experiences of psychological and physical aggression (Table 4). Aditionally, witnessing three or more acts of violence at home was also associated with experiences of bullying (AOR: 2.07, 95%CI: 1.17–3.67) and cyberbullying (AOR: 3.74, 95%CI: 1.76–7.96).

**Table 4:** Associations (odds ratio and 95% confidence interval) of witnessing violence at home with aggressive behaviors and depressive symptoms

	Physical fights		Bullying		Cyberbullying		Depressive symptoms	
	OR (95%CI)	AOR* (95%CI)	OR (95%CI)	AOR* (95%CI)	OR (95%CI)	AOR* (95%CI)	OR (95%CI)	AOR* (95%CI)
Number of witnessed acts of violence at home								
0	1 (ref.)	1 (ref.)	1 (ref.)	1 (ref.)	1 (ref.)	1 (ref.)	1 (ref.)	1 (ref.)
1–2	**2.51 (2.05–3.08)**	**2.01 (1.53–2.64)**	**1.90 (1.56–2.33)**	1.31 (0.99–1.72)	**2.07 (1.44–2.97)**	1.19 (0.71–1.99)	**2.74 (2.19–3.43)**	**1.97 (1.43–2.72)**
≥ 3	**4.93 (3.29–7.38)**	**3.33 (1.88–5.92)**	**2.87 (1.91–4.33)**	**2.07 (1.17–3.67)**	**5.63 (3.29–9.63)**	**3.74 (1.76–7.96)**	**3.81 (2.45–5.93)**	**2.22 (1.11–4.44)**

*Adjusted for child´s sex, maternal education, parental unemployment, family structure and child’s experience of psychological and physical aggression

## Discussion

Our findings revealed that children who had witnessed violence at home are more likely to be involved in physical fights, bullying and cyberbullying behaviours, as well as, to develop relevant depressive symptomatology at 13 years of age, and this association is independent of sex, socioeconomic circumstances, family structure and self-experience of psychological and physical aggression.

Our results are aligned with previous studies also reporting an association between exposure to violence in the domestic environment and violent behavior among adolescents (Duman & Margolin, 2007; Holt et al., 2008). This relationship can be explained by Social Learning Theory, which suggests that children may model the aggressive behaviors they observe and consider them as the proper way to deal with conflicts (Bandura, 1973), thereby hindering their ability to establish healthy relationships in the future. Kitzmann et al. (2003) suggested that while exposure to domestic violence was associated with increased aggression, other factors, such as being physically abused, may also play important roles in the development of aggressive behaviors (Kitzmann et al., 2003). However, in our study, the association remained statistically significant even after adjusting for the child’s experience of psychological and physical abuse, supporting that witnessing violence can be as harmful as directly experiencing abuse.

Furthermore, in our results, the witnessing of violence at home was associated with involvement in cyberbullying but only in the most severe cases, where children reported to have witnessed three or more acts of violence at home. Previous literature looking at this association gives contradictory results. Although a recent study reported a positive association between exposure to domestic violence and cyberbullying among secondary school students (Chen et al., 2023), a study conducted by Vieira et al. did not find a significant association between exposure to domestic violence and cyberbullying victimization among adolescents (Vieira et al., 2019). They argued that cyberbullying may be more influenced by peer dynamics and online social contexts than by experiences within the home environment (Vieira et al., 2019).

Our findings regarding sex differences in exposure to violence at home are inconsistent with previous literature which suggests that girls are just as likely or more likely to report witnessing domestic violence than boys (Hietamäki et al., 2021). Some authors argue that both boys and girls are affected in the same way but they may differ in the way they respond to these adverse events (Forke et al., 2018). In terms of behavioral outcomes, our study found that boys were more likely to be involved in physical fights and engage in cyberbullying, particularly as perpetrators. In contrast, girls were more often involved in bullying, as victims, and reported higher levels of depressive symptoms. This pattern is well documented in the literature and reflects broader developmental and psychosocial trends (Buckner et al., 2004; Holt et al., 2008). Notably, the associations between witnessing domestic violence and involvement in physical fights, bullying, and depressive symptoms were statistically significant regardless of the child’s sex. These results contrast with the majority of existing literature, which often reports distinct gendered patterns in how adolescents respond to violence (Forke et al., 2018). Furthermore, the sex of the perpetrator also plays a significant role in shaping adolescents’ responses to violence. Research shows that witnessing a male caregiver commit violence in the home increases boys’ likelihood of perpetrating violence in their own adolescent relationships, while for girls, it heightens the risk of becoming both victims and perpetrators. Adolescents who witness a female caregiver perpetrate violence also face a greater risk of being both victims and perpetrators of relationship violence (Forke et al., 2018). Although our study does not allow us to determine the sex of either the perpetrator or the victim, identifying the sex of the adult perpetrator may be important when assessing cases of family violence. In particular, domestic violence and gender-based violence are related but not equivalent concepts, and a proportion of the violence witnessed by adolescents in the home may reflect gender-based violence dynamics. As our data did not include information on the sex of the perpetrator or the nature of the relationship between the adults involved, it was not possible to distinguish gender-based violence from other forms of domestic violence. This should be considered when interpreting the findings, as gender-based violence may have specific implications for adolescents’ health and well-being.

Beyond sex, other sociodemographic factors played a significant role in the associations explored in our study. Lower parental education, unemployment, lower household income, and family structures without both parents were associated with higher rates of violence at home and involvement in aggressive behaviors. These findings corroborate previous research, who identified similar socioeconomic risk factors for exposure to domestic violence and subsequent aggressive behaviors in adolescents (Carlson et al., 2019). Although domestic violence occurs across all socioeconomic groups, research indicates that poor families are disproportionately affected (Buckner et al., 2004). However, some authors argue that while socioeconomic factors are important, they may not fully explain the complex relationship between domestic violence exposure and child outcomes, suggesting the need to consider additional mediating and moderating factors (Margolin & Gordis, 2000). As a previous study demonstrated, exposure to violence in the family increases adolescent involvement in violent behavior by fostering associations with violent peers, approval of violent norms, and negative emotions. These mediating effects remained significant after controlling for gender and age, while the direct effect of family violence was small and only marginally significant (Li et al., 2021).

Additionally, although exposure to violence at home can have long-term consequences, including increased risk for depression, anxiety and post-traumatic stress disorder, difficulties in emotion regulation and reduced life satisfaction in adulthood (Pang & Thomas, 2020), it is important to avoid overly deterministic interpretations of these findings. Not all children exposed to violence at home go on to develop psychological or behavioural difficulties; many demonstrate significant resilience (Holt et al., 2008). Some children and adolescents emerge from such experiences with minimal psychological harm or even strengthened coping and survival skills (Holt et al., 2008; Kitzmann et al., 2003). A secure attachment to a non-violent parent or other significant caregiver is one of the most consistently identified protective factors, contributing to emotional stability and reduced trauma-related distress (Graham-Bermann et al., 2006). Additionally, self-esteem, a foundational element of resilience, plays a critical role in helping children develop effective coping strategies (Martin, 2002). Children who perceive themselves as having agency and maintain a positive self-concept may be better equipped to withstand the psychological impact of domestic violence (Holt et al., 2008). Collectively, these findings emphasize the diverse ways in which children respond to violence exposure and highlight the critical role of supportive relationships and protective psychosocial factors in fostering resilience.

Early intervention programs targeting families at risk may be critical to preventing the intergenerational transmission of violence. Schools and community-based initiatives should incorporate violence prevention strategies, including conflict resolution training, emotional regulation techniques, and resilience-building interventions, to mitigate the psychological and behavioural impact of adolescents exposed to violence at home. Policymakers should prioritize the development and implementation of comprehensive policies aimed at reducing domestic violence and supporting affected families. This includes increasing funding for domestic violence shelters, expanding access to counselling and mental health services, and launching educational campaigns to raise public awareness about the long-term effects of domestic violence on children. Additionally, raising awareness among healthcare professionals is essential to improve the early identification of at-risk children and to ensure timely referral to appropriate support services.

This study presents several strengths that enhance its contribution to the understanding of the relationship between exposure to violence at home and adolescent involvement in aggressive behaviours. It draws on data from the Generation XXI cohort, a population-based birth cohort from the Porto metropolitan area in Portugal, providing a substantial and socio-demographically diverse sample. This enhances both the statistical power and the generalizability of the findings within the study population. Moreover, the study assesses five distinct types of violent acts witnessed at home, allowing for a nuanced understanding of the nature and severity of violence exposure. By examining three forms of adolescent aggression—physical fighting, bullying, and cyberbullying—the study captures a broad spectrum of aggressive behaviours relevant to contemporary adolescent social contexts. The inclusion of multiple covariates strengthens the internal validity of the findings by accounting for potential confounding variables that may influence the association between domestic violence exposure and aggressive behaviours. Nonetheless, several limitations should be acknowledged. The use of self-reported measures may introduce information bias, as respondents might underreport or overreport experiences due to social desirability or recall limitations. Another relevant aspect pertains to the questions used to assess exposure to this form of violence. The current methodology does not enable the assessment of key conceptual criteria that define bullying, such as repetition, intentionality, and power imbalance. Similarly, these measures do not allow for a precise identification or classification of victims or perpetrators according to established definitions of bullying. Nevertheless, it can be inferred that, if the child attributes significance to the experience, it is likely to have left a lasting impact on their development or well-being. Furthermore, the cross-sectional design precludes causal inference, and longitudinal analyses are necessary to establish temporal relationships and assess the long-term effects of domestic violence on individuals’ outcomes. Additionally, the use of a general question, rather than a standardized instrument, to assess bullying and cyberbullying may have led to an underestimation of involvement, as it relies on participants’ own interpretation of these concepts (Brochado et al., 2021). For analytical purposes, we dichotomized these variables due to limited statistical power and to ensure consistency with the measurement approach. However, this approach was consistent with the measurement strategy used for physical fighting, ensuring comparability across the different forms of aggression assessed. A further limitation is that our measure of violence captured adolescents’ reports of witnessing violence between adults at home, without information on the sex of the perpetrator or the relationship between those involved. Therefore, we were not able to distinguish gender-based violence from other forms of domestic violence.

Finally, it is important to acknowledge that our study did not examine child-to-parent violence, an emerging form of family violence with potential emotional, behavioral, health and social consequences for adolescents and their families (Martínez-Ferrer et al., 2020; Suárez-Relinque et al., 2023). Incorporating this dimension in future research could provide a more comprehensive understanding of household violence dynamics and their impact on adolescent health and well-being.

In violence research, the terms “children who experience violence” or “children exposed to domestic violence” are preferred over “witnessing violence” to challenge the passive framing of childhood, which can hinder the recognition of children as direct and equal victims in these contexts of violence. Our findings showed significant effects of witnessing violence at home on children’s health, supporting the notion that such violence impacts children directly, rather than being an issue confined to the household and assumed to have no direct effect on them. Also, looking at children as direct and equal victims will allow us to focus on providing a more effective legal and safeguarding framework for children.

## Conclusion

This study provides evidence of an association between witnessing violence at home and the emergence of aggressive behaviours and depressive symptoms during adolescence. The findings underscore the complex interplay of sociodemographic factors in shaping these outcomes and reinforce the urgent need for targeted interventions and policies to prevent domestic violence and mitigate its harmful effects on children. Further longitudinal research is essential to clarify the causal pathways involved and to inform the development of effective, evidence-based strategies to break the intergenerational cycle of violence.

## Data Availability

The data from Generation XXI are not publicly available due to privacy or ethical restrictions. The data can be made available for research proposals on request to the Generation XXI Executive Committee (generationxxi@ispup.up.pt). Further information about Generation XXI can be obtained via the Generation XXI website [www.geracao21.com] or by emailing generationxxi@ispup.up.pt
